# TCR sequencing: applications in immuno-oncology research

**DOI:** 10.1016/j.iotech.2023.100373

**Published:** 2023-02-04

**Authors:** Á.F. Sanromán, K. Joshi, L. Au, B. Chain, S. Turajlic

**Affiliations:** 1 Cancer Dynamics Laboratory, The Francis Crick Institute, London, UK; 2 Department of Medical Oncology, The Royal Marsden NHS Foundation Trust, London, UK; 3 Renal and Skin Unit, The Royal Marsden NHS Foundation Trust, London, UK; 4 Department of Medical Oncology, Peter MacCallum Cancer Centre, Melbourne, Australia; 5 Cancer Immunology Program, Peter MacCallum Cancer Centre, Melbourne, Australia; 6 Sir Peter MacCallum Department of Oncology, The University of Melbourne, Australia; 7 Division of Infection and Immunity, University College London, London, UK; 8 Department of Computer Science, University College London, London, UK; 9 Melanoma and Kidney Cancer Team, The Institute of Cancer Research, London, UK

## Abstract

• T-cell receptor (TCR) interaction with major histocompatibility complex–antigen complexes leads to antitumour responses.
• TCR sequencing analysis allows characterisation of T cells that recognise tumour neoantigens.
• T-cell clonal revival and clonal replacement potentially underpin immunotherapy responses.

## Introduction

Immune checkpoint blockade (ICB)^1^ and other immunotherapies designed to enhance effective antitumoural T-cell responses^2^ have revolutionised cancer care, thus placing characterisation of T-cell responses as a central piece in immuno-oncology research. In recent years, T-cell receptor (TCR) sequencing has emerged as a powerful tool to characterise the breadth and depth of T-cell responses. This review will describe the biology of TCRs and methods that capture TCR sequences, and summarise the literature linking TCR sequence characteristics to antitumoural T-cell responses in patients treated with ICB. Finally, we discuss the limitations of current TCR sequencing approaches and areas for future development.

### Biology of T-cell receptors

The TCR, originally described by Tak Wah Mak,^3^ is a heterodimeric receptor found on the surface of all T cells; in humans, the majority (95%) of TCRs are composed of an alpha and beta chain (αβ TCRs), and the remaining 5% are made of a gamma and delta chain (γδ TCRs). The key characteristic of these receptors—particularly of αβTCRs—is their vast sequence diversity. Each individual expresses an estimated 10^8^-10^9^ distinct TCRs,^4^^,^^5^ produced by a unique and continuous process of somatic recombination during development and differentiation of T cells in the thymus. The diversity of T cells and their corresponding TCR sequences generated by this process is critical to increase the probability that at least one of the T cells will recognise an unseen antigen, helping to mount a rapid and effective immune response, especially to pathogens. In this context, a ‘T-cell clone’ refers to a lineage of T cells with identical TCRs.

TCR sequence diversity is afforded by differences in the complementarity determining regions 1, 2, and 3 (CDR1, CDR2, CDR3), made up of short protein loops forming a binding surface for the target antigen. αβTCRs recognise short peptides produced by endogenous or exogenous antigen processing and bound within the binding groove of the major histocompatibility molecules (MHC) (peptide–MHC complex; pMHC). The most variable CDR3 regions of the TCR interact directly with the peptide, and hence are key in determining antigen specificity of individual T-cell clones.

Specific antigen recognition is fundamental to T-cell activation. The process of T-cell activation is reviewed in detail elsewhere.^6^ Briefly, when the TCR binds to a target MHC–peptide complex, the TCR-associated CD3 complex, comprising six chains (two ε chains, one δ chain, one γ chain, and two ζ chains), is phosphorylated at the intracellular immunoreceptor tyrosine-based activation motif (ITAM) domains by both Lcr and CD45-associated Fyn kinases. Zap70 then binds to the phosphorylated CD3 ζ. The T-cell co-receptor (CD4 or CD8) now also binds to the pMHC complex via a separate binding region. This brings the Lck molecule close to Zap70 to trigger further signalling cascades via phosphorylation. The end result of T-cell activation is the transcription of multiple genes that promote T-cell differentiation and proliferation of antigen-specific T cells. Intense proliferation means a high number of T cells will share the same genetic ancestry (i.e. share the same lineage). The general term which defines this process is ‘T-cell clonal expansion’.

TCR sequencing enables accurate and reproducible estimation of the relative frequency of different TCRs in blood or tissue, defined as the ‘TCR repertoire’. Therefore, analysis of the TCR repertoire provides a window into the nature of the adaptive immune response for an individual.

### TCR sequencing methods

There are several methods to profile pooled T-cell populations referred to as ‘bulk’ TCR sequencing. Bulk TCR sequencing enables the study of TCR diversity and clonality, and allows comparison of repertoires between samples based on the frequency of single TCR chains.

Currently available bulk TCR sequencing protocols can use either gDNA or RNA as starting material (derived from fresh or formalin-fixed samples) with pros and cons to each approach.^7^ The variability, diversity, and joining (VDJ) complex represents a minute portion of the total gDNA. Therefore, if gDNA is used as the starting material, rare TCR sequences are less likely to be detected. This potentially results in a more restricted view of the TCR repertoire. The use of RNA helps to overcome this as there are multiple copies of the same RNA molecule within an individual cell. This increases the likelihood of less-frequent TCR sequences being detected, and allows to quantify the mRNA expression of the TCR sequence, which is not achievable when using gDNA. Further, introns and possible residual VDJ segments not used in somatic recombination may act as binding sites for primers when the gDNA template is used, increasing sequencing bias, risk of background amplification, and sequencing errors. On the other hand, RNA requires more careful handling, given risk of degradation.

**Figure 1:**
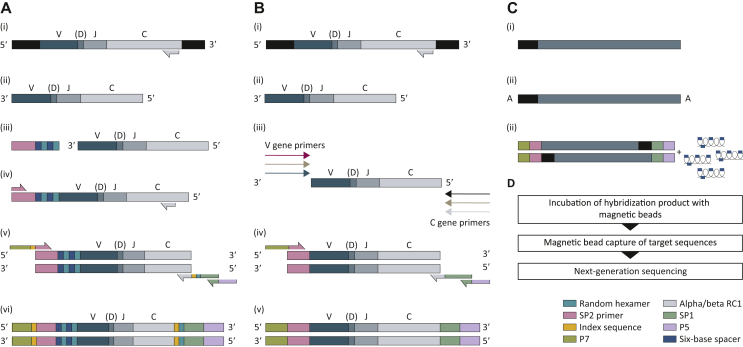
**Schematic representation of bulk TCR sequencing library preparation methods.** (A) 5′RACE PCR with incorporation of a UMI. (i) RNA extraction from cells or tissues. (ii) Reverse transcription of RNA to generate cDNA. (iii) Single-stranded cDNA ligation of a UMI. (iv) PCR1. (v) PCR2. (vi) Prepared TCR libraries are analysed using next-generation sequencing platforms. (B) Multiplex PCR using RNA. (i) RNA extraction from cells or tissues. (ii) Reverse transcription of DNase-treated RNA to generate cDNA. (iii) PCR1 using all known primers for TCR variable and constant region primers. (iv) PCR2. (vi) Prepared TCR libraries are analysed using next-generation sequencing platforms. (C) Target enrichment. (i) End-repair of RNA. (ii) A-tailing. (iii) Following adaptor ligation, prepared libraries are incubated with bespoke RNA library baits complementary to V, J, and C gene segments. (iv) The hybridisation product is incubated with magnetic beads and a magnet is used to capture target sequences. Prepared TCR libraries are analysed using next-generation sequencing platforms. 5′RACE, 5′ rapid amplification of complementary DNA ends; TCR, T-cell receptor; UMI, unique molecular identifier.

Bulk TCR library preparation is achieved through one of three approaches: (i) multiplex PCR, (ii) targeted in-solution enrichment, and (iii) 5′ rapid amplification of complementary DNA ends (5′RACE) PCR (Figure 1).

Multiplex PCR methods amplify the TCR CDR3 region, by combining primers for all known TCR variable (V) and joining [J; for genomic DNA (gDNA)] or constant [C; for RNA/complementary DNA (cDNA)] regions. A key limitation is amplification bias leading to misrepresentation of the relative abundance of TCR genes, ultimately impacting TCR clonality read-out. There are several approaches to reduce amplification bias including adjustment of primer concentration,^8^ or incorporation of unique molecular identifiers (UMIs).^9^

Targeted enrichment involves fragmentation of gDNA or purification of mRNA followed by end-repair, A-tailing, and adapter ligation. Target sequences are then enriched with bespoke RNA baits complementary to V, J, and C gene segments followed by target DNA hybridisation.^10^^,^^11^

5′RACE PCR requires reverse transcription of RNA to cDNA. Then, there is a two-step amplification reaction of cDNA using two different primer pairs. In this method,^12^ the anchor sequence for the reverse primer is introduced into the first cDNA strand, either by incorporation of a switch oligo^12^ or by single-stranded ligation.^13^ The forward primer is located at the 5′ end of the constant region gene, thus restricting the method to RNA-based templates. Next, nested PCRs (using primers targeting a small region of the amplification product from the first PCR) introduce sequencing adaptors and primer sequences for the amplification reaction. 5′RACE PCR has important advantages;^14^ it does not require optimisation of multiple V region primers, so it is less prone to bias and it enables the introduction of UMIs at the reverse transcription stage, which can be used for correction of sequencing errors and PCR bias.

The result from bulk TCR sequencing methods is a set of unpaired TCRα and TCRβ chains from different T cells. The unpaired nature of the data generated limits the resolution of TCR clonality and diversity estimations because it hinders the distinction between T-cell clones with the same TCRα chain paired to distinct TCRβ chains (or vice versa—with the same TCRβ chain but paired to different TCRα chains).^15^, ^16^, ^17^ This also hinders accurate inference of TCR antigen specificity because antigen recognition strongly relies on the combination of the TCRα and TCRβ chains. Fortunately, various methods have recently been developed to optimise re-pairing of TCRα and TCRβ chains from bulk TCR sequencing. Howie et al.^18^ used a combinatorics-based library preparation (pairSEQ) where T cells are randomly distributed so that each well contains a low number (10-100s) of T cells, which will be tagged with the same well-specific DNA barcode. The key of this method is that T cells from the same clone (hence sharing the same pair of TCRα and TCRβ chains) fall in different wells. After pooling and sequencing, each TCRα and TCRβ chain will have an associated set of well barcodes. TCRα and TCRβ chains that are paired are part of the same TCR and hence necessarily have fallen in the same wells during library preparation and consequently share the same set of well barcodes. Therefore, bioinformatic reconstruction of TCRα–TCRβ pairs is possible by matching TCRα and TCRβ chains linked to the same set of well-specific barcodes. A potential shortcoming of this method is its incapacity (i) to pair chains of dual TCRα and TCRβ receptors (i.e. TCR dimers of only α or β chains, respectively,^19^^,^^20^) and (ii) to pair TCRα and TCRβ chains when one of the two chains is the same in two different TCR receptors. Lee et al.^21^ proposed a solution to both problems by developing a computational algorithm based on a repeated sampling strategy to flexibly infer TCR pairs from data generated by an updated library preparation version of pairSEQ.

Despite these novel developments, the current optimal way to pair TCRα and TCRβ chain is through single-cell TCR sequencing (scTCR-Seq). scTCR-Seq approaches generally rely on the adaptation of bulk 5′RACE approaches (described earlier), and different reviews cover in greater depth the details of this methodology.^22^^,^^23^ The additional advantage of single-cell TCR sequencing is its high accuracy in the estimation of TCR clonality and diversity in comparison to bulk TCR sequencing.^13^

An alternative to dedicated targeted single-cell TCR sequencing is the computational reconstruction of TCR sequence from full-length single-cell RNA sequencing data. Computational methods to extract the TCR sequence are generally based on a combination of alignment and *de novo* assembly of TCR reads,^20^^,^^24^^,^^25^ allowing linkage of inferred TCR sequences with T-cell transcriptional profiles. The limitation of this approach is its lower accuracy, given the strong dependence on the number of captured TCR mRNA molecules during library preparation, which is determined by the sequencing depth and TCR expression levels on each cell.^22^ Dedicated paired single-cell TCR and RNA sequencing circumvents this problem.

Collectively, single-cell sequencing platforms are currently limited by cost, number of cells processed, and requirements for high-quality (i.e. fresh) input material. In contrast, bulk TCR sequencing is considerably cheaper, thus scalable for the number of T cells profiled, and suitable for profiling of formalin-fixed paraffin-embedded (FFPE) tissue.^22^

### Using TCR repertoires to study adaptive immune recognition in cancer

The tumour microenvironment (TME) is frequently infiltrated by a variety of T cells [tumour-infiltrating lymphocytes (TILs)], most of which are not tumour-reactive (so-called bystander T cells).^26^ Identifying which of the many T cells in the TME recognise tumour antigens is a long-standing quest. In principle, antigen specificity could be determined from TCR sequence. However, the rules relating TCR sequence to antigen specificity are incompletely understood. The mapping of antigens to TCRs is one to many, and many to one, i.e. the same TCR can recognise more than one peptide, and multiple TCRs can recognise the same peptide.^27^ Inferring antigen specificity from TCR sequence is even more difficult in CD4+ TCRs because they usually show higher promiscuity than CD8+ TCRs. Advanced computational methods are in development to predict pMHC antigen specificity of individual TCRs from the TCR sequence alone. To date, computational studies have approached this by training machine learning and deep learning models with sequence and/or structural features from available TCR sequence–antigen pairs.^15^^,^^28^, ^29^, ^30^, ^31^, ^32^, ^33^, ^34^, ^35^ The hope is that these algorithms will enable the prediction of cognate pMHC of any T cell with only its TCR sequence as input. Despite the intense recent efforts in this field, the accuracy of computational TCR specificity prediction remains poor. Improved algorithm accuracy is needed before use in immuno-oncology translational research studies.

TCR specificity can also be determined using functional assays. Peptide–MHC multimers can be used to identify tumour T cells that exhibit high affinity for the multimer. Commonly used MHC multimers are tetramers conjugated with fluorochromes to enable the identification and isolation of antigen-reactive T cells using flow cytometry. Such workflows depend on reliable MHC-binding predictions and the generation of specific MHC alleles. Traditionally, this was limited to only human leukocyte antigen (HLA)-A∗0201, but it is now possible to study T-cell specificity against antigens presented by a wide range of HLA class I alleles.^36^

A common application of peptide–MHC multimers is the identification of the TCRs that recognise tumour neoantigens in a given patient. To do so, putative tumour-derived neoantigens are first inferred by applying bespoken algorithms to sequencing data.^37^ Based on these neoantigens, a battery of patient-specific peptide–MHC multimers is then generated and used to identify which T cells recognise them; these are the reactive TILs. Further determination of the TCR sequencing of these T cells is possible using downstream TCR sequencing. However, these functional assays remain challenging, labour intensive, and costly to conduct. Another major bottleneck is the accuracy of the algorithms predicting tumour neoantigens from sequencing data.^37^ This limits the current application of these approaches to translational studies.

**Figure 2:**
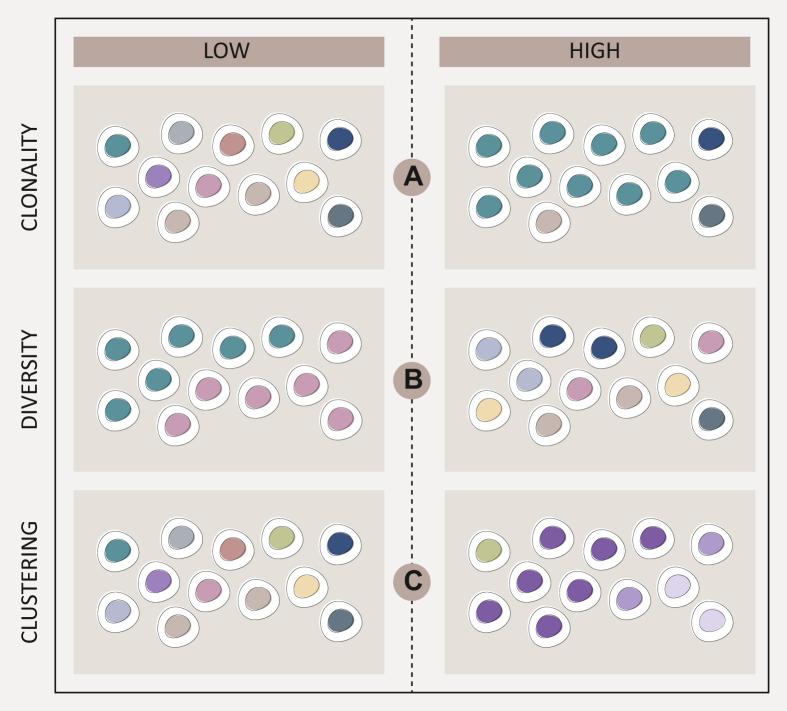
**Schematic representation of TCR metrics in immuno-oncology measured by TCR sequencing.** In this schema, different colours of T cells represent different clones; same colour in different shades represent more closely related T-cell clones, compared to different colour T cells. (A) Low TCR clonality is where a low number of T cells share the same genetic ancestry (and therefore TCR sequence) and indicate lack of T-cell expansion, whereas high TCR clonality is where a high number of T cells share the same genetic ancestry (and therefore TCR sequence), suggesting T-cell activation, proliferation, and expansion have occurred in response to a cognate antigen. (B) Low TCR diversity is where there are few T cells with distinct TCR sequences within a population, whereas high TCR diversity is where a high number of T cells with distinct TCR sequences are detected within a population. (C) Low TCR clustering is where there is limited similarity (unrelated CDR3 peptide sequences) between T cell(s) within a population, whereas high TCR clustering is where groups of similar (closely related CDR3 peptide sequences) T cells exist within a population. TCR, T-cell receptor.

A simpler, though indirect compared to functional assays, way to study antitumour T-cell responses is the quantification of TCR repertoire diversity, clonality, and clustering, given how all of these metrics are related to T-cell activation and proliferation (Figure 2). Highly clonal TCR repertoire is generally assumed to imply antigen-triggered expansion from a limited number of T-cell clones. This is because activation and proliferation of a T-cell clone following cognate antigen stimulation increases the relative frequency of its TCR sequence through clonal expansion. In contrast, highly diverse TCR repertoires are taken as evidence that the clones either have not expanded or multiple clones have undergone antigen-triggered expansion with similar intensities. The clonal expansions underlying each of these scenarios are non-existent and weak, respectively, and hence the general assumption is that T cells in both cases are non-reactive. However, TCR clonality metrics are imperfect at providing a definitive read-out for antitumoural T-cell responses. This is because T-cell expansion leading to high TCR clonality can arise from sources distinct to tumour-specific responses, for example, from exogenous viral antigenic stimulation. Besides clonality and diversity metrics, TCR sequence similarity measured as clusters (formed by grouping TCRs with similar CDR3 peptide sequences) and networks (formed by grouping closely related TCR clusters) also provide insight into the antigenic specificity of TCRs.^38^, ^39^, ^40^, ^41^ Numerous studies^15^^,^^28^^,^^42^ have shown that epitope-specific TCR repertoires comprise of clusters of TCRs that share core sequence similarities, particularly within the CDR3 loop.^43^^,^^44^

By analysing these TCR sequencing metrics derived from tumour samples, we have recently reported the presence of clusters and networks of related TCR sequences within populations of TILs^45^ in non-small-cell lung cancer (NSCLC), indicating T-cell antigen specificity.^15^ This was demonstrated using TCR sequencing combined with MHC–peptide multimer sorting and single-cell RNA sequencing (scRNA-Seq) on multi-region tumour samples and adjacent non-tumour tissue obtained from patients with early-stage NSCLC undergoing surgery. Further, by linking TCR and RNA sequencing data, studies in melanoma^46^ and gastrointestinal cancers^47^ had used functional assays to show that tumour-neoantigen-specific T cells have a different transcriptional state compared to non-neoantigen-specific T cells. Caushi et al. reached a similar conclusion in NSCLC.^48^ Hanada et al.^49^ recently went one step further by deriving a gene-expression signature that enables the rapid identification of CD4 and CD8 neoantigen-reactive TCRs in NSCLC. Common transcriptional markers of neoantigen-specific T cells in all these studies are low expression of IL-7R and the high expression of CXCL13—the latter was the strongest gene-expression bulk RNA-Seq-based ICB biomarker in a recent meta-analysis.^50^ Studies to date suggest a convergence in the transcriptional profile of the neoantigen-specific T cells across tumour types. This is remarkable considering the vast difference in the overall immune compositions of different tumour types, and it also suggests that a pan-cancer transcriptional signature can discriminate between tumour infiltration by tumour-reactive versus bystander T cells. Lowery et al. recently reported a 243-gene signature that identified neoantigen-specific CD8+ T cells using 10 metastatic cancers of different histologies.^51^ Broader validation of this signature will be critical.

### Using TCR repertoires to understand the principles of ICB response

#### Source of T cells driving clinical response to ICB

**Figure 3:**
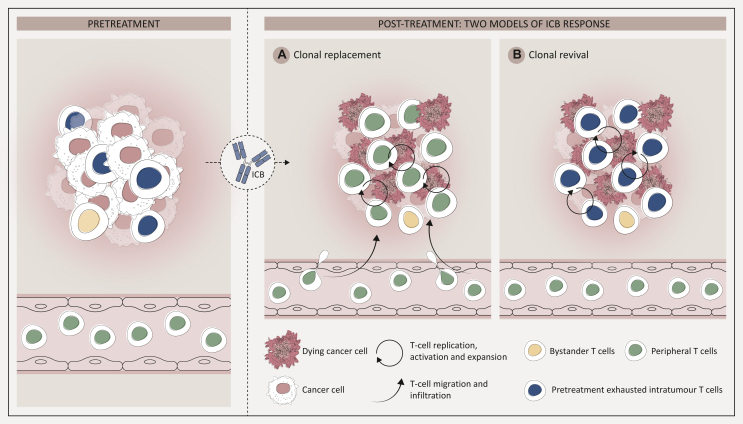
**Clonal replacement and clonal revival models of response to ICB**. The pre-treatment with the ICB TME with TILs and associated vasculature is shown. ICB application leads to T-cell replication, activation, and expansion which leads to tumour cell killing by T cells. This is explained as follows: (A) the T-cell clonal replacement model of ICB response suggests pre-existing TILs are replaced by novel T cells targeting tumour-specific antigens which have trafficked into the TME; and (B) the T-cell clonal revival model of ICB response suggests reinvigoration of pre-existing TILs are responsible for cancer cell killing. Different colours of T cells represent different clones. ICB, immune checkpoint blockade; TILs, tumour-infiltrating lymphocytes; TME, tumour microenvironment.

Whether the key mechanism underpinning ICB response is through driving novel T cells into tumours (termed ‘T-cell clonal replacement’) or reinvigoration of pre-existing TILs (termed ‘T-cell clonal revival’) is under debate (Figure 3).^52^ Multiple studies have leveraged longitudinal TCR sequencing of tumour samples under ICB treatment to explore these hypotheses. From pre-treatment biopsies, pre-existing antitumour adaptive immunity is indicated by high TCR clonality and increased TCR clusters. Repeated post-treatment tumour biopsies allow inference of whether the tumour-infiltrating T cells are pre-existing or novel: post-treatment TCR repertoires that are similar to pre-treatment would indicate maintenance of pre-existing T-cell clones, while distinct TCR repertoires detected after treatment would indicate that novel T-cell clones have replaced pre-existing ones. If data on peripheral TCR repertoires (i.e. from blood contemporaneously sampled with tumour) are available for comparison, inferences on tumour-extrinsic sources of novel T-cell clones can be made. Tumour-derived TCR sequencing data can be complemented by paired RNA sequencing to reveal transcriptional phenotypes of relevant T-cell populations.

Yost et al.^53^ recently associated clonal replacement as a key ICB mode of action. They analysed 15 patients with advanced basal cell or squamous cell carcinoma following programmed cell death protein 1 (PD-1) blockade, integrating scRNA-Seq and TCR-Seq on paired pre/post-treatment tumour biopsies. They found the sequences of significantly expanded TCR clones after treatment were not detectable before treatment. The expanded T cells were therefore ‘novel’, and transcriptome data associated post-treatment T cells with an exhausted phenotype. Analysis from a five-patient subset showed 11.8% and 35.5% of these novel TILs were detectable in blood before and after treatment, respectively. The authors posited that anti-PD-1 promotes infiltration of CD8 T cells from blood to tumour, by inducing tumour recognition to antigens distinct to those recognised by expanded TCR clones at baseline. Subsequently, a study on four patients (one with lung cancer; three with renal cancers) with matched tumour/blood samples at baseline (single timepoint) showed blood and tumour compartments extensively shared expanded clonal lineages of T effector cells.^54^ This suggests blood-expanded TCR clones have a role in tumour infiltration. However, the same report^54^ reanalysed longitudinal samples from two patients in the cohort of Yost et al.^53^ and showed that for post-treatment intratumoural T cells, ‘non-exhausted’ clones were more likely to be blood-associated, whereas ‘exhausted’ clones were more likely to be blood-independent (i.e. they were possibly pre-existing intratumoural). This suggests that ICB introduces novel tumour-infiltrative TCR clones which expand but may not have T-cell exhaustion features.

Further evidence in NSCLC highlights clonal replacement as a key ICB mode of action. Zhang et al. analysed longitudinal blood, tumour, and normal lung samples from 21 patients with stage I-III NSCLC treated with neoadjuvant anti-PD-1.^55^ They found that patients with <10% residual tumour after treatment [termed ‘major pathological response’ (MPR)] had ∼80% of the intratumoural and most expanded TCR clonotypes shared with peripheral blood compared to patients without MPR in their tumours. Further, greater expansion of peripheral T-cell clones correlated with MPR, suggesting ICB response is linked to remodelling of the blood TCR repertoire and trafficking of T cells into tumours. Caushi et al.^48^ analysed tumour and blood samples from 20 NSCLC patients, also treated with neoadjuvant anti-PD-1. They used an *in vitro* assay to directly test reactivity of expanded intratumoural and peripheral TCR clones against tumour neoantigens, and identified the neoantigen-specific T cells and their corresponding TCR sequences. Intriguingly, neoantigen-specific TCRs were detected in the peripheral blood in most patients but only in a third of tumour samples. However, detection of these TCRs did not correlate with ICB response in this study.^48^

Collectively, clonal replacement postulates that ICB triggers tumour infiltration of novel CD8+ T cells, replacing pre-existing CD8+ T cells that are incapable of cancer killing, presumably due to chronic activation and exhaustion. However, these studies describing dynamic TCR repertoire changes did not consistently show correlation with clinical responses to ICB. Additionally, while the source of novel TCR clones may be tumour-extrinsic (i.e. from blood, but theoretically also from local lymph nodes or tumour periphery), tumour-intrinsic sources (i.e. expansion of rare pre-existing clones) cannot be excluded.

Clonal revival proposes an alternate view in that ICB clinical response derives from pre-existing (i.e. tumour antigen primed) intratumoural T cells capable of cytotoxic reinvigoration despite displaying features of exhaustion. We have recently investigated this using paired longitudinal tumour and blood samples from patients with metastatic clear-cell renal cell carcinoma in a phase II study of first-line anti-PD-1 (nivolumab).^56^ In bulk tumour samples, we showed that responders to nivolumab had significantly higher pre-treatment intratumour TCR clonality and cluster structure than non-responders, suggesting pre-existing adaptive immunity. Combining bulk and single-cell TCR and RNA-Seq data, we observed both novel and maintenance of pre-existing TCR clones in post-treatment samples—but only the latter correlated with ICB response.^56^ Moreover, following treatment administration, we observed anti-PD-1 binds to both novel and pre-existing CD8+ T cells, but only pre-existing expanded TCR clones in responders had a cytotoxic phenotype (i.e. up-regulation of GZMB/K) and not in novel clones or in non-responder patients.^56^ This suggests ongoing antigen-driven stimulation of pre-existing T cells exclusively in responders, and ICB-facilitated maintenance and cytotoxic reinvigoration of these clones. These findings were not recapitulated in blood, which had lower TCR clonality scores overall compared to tumour and did not correlate with anti-PD-1 response. Supporting this finding was our reanalysis of the longitudinal data from the Yost et al. publication.^53^ We specifically identified the expanded TCRs before treatment to track them in the post-treatment samples. This showed a trend for increased maintenance of expanded pre-existing clones in responders (*P* value = 0.08).^56^

In other tumour types, Valpione et al. analysed pre-treatment biopsies from 20 patients with metastatic melanoma undergoing anti-PD-1 treatment.^57^ They found higher TCR clonality pre-treatment in patients responding to ICB. This finding was validated in baseline biopsies from an external cohort of metastatic melanoma patients (*n* = 106) treated with anti-PD-1 or sequential anti-PD-1/anti-cytotoxic T-lymphocyte–associated antigen 4 (CTLA-4).^58^ In breast cancer, Bassez et al. carried out single-cell analysis of 29 patients treated with neoadjuvant anti-PD-1,^58^ although correlation with clinical outcomes could not be assessed due to short study follow-up. They reported that one-third of the tumours harboured T cells expressing anti-PD-1, and that these cells appeared at a significantly higher frequency in post-treatment samples, consistent with intratumoural clonal expansion upon ICB administration.

One potential way to reconcile apparently contradicting reports supporting clonal replacement with those supporting clonal revival is that the mechanism underpinning ICB response is context-dependent and hence can change, for example, in distinct tumour types. However, contradicting data exist even within the same tumour type. Contrary to the aforementioned reports supporting clonal replacement in NSCLC,^48^ Liu et al. applied paired scRNA-Seq and TCR-Seq to 47 tumour biopsies from 36 NSCLC patients receiving ICB and described the accumulation of exhausted T cells in treatment-responsive tumours likely to be derived from both peripheral T cells and local expansion of pre-existing T cells.^59^

Another explanation to these divergent read-outs is the existence of confounding factors unequally controlled in different studies: differences related to (i) tumour sample characteristics (such as primary versus metastatic tumour sampling, size of tumour samples and thus scope for TCR capture, and inherent spatial TCR heterogeneity^45^^,^^56^^,^^60^^,^^61^), and (ii) timing of on-treatment sampling, ICB regimens, and ICB dosage. Further, we have recently detected heterogeneity in the TCR sequences, clonality and diversity inferred from bulk RNA-Seq in spatially separate biopsies of the same tumour, supporting that currently published data may suffer from sampling bias.^56^ In most studies, tumour-derived TCR sequences refer to the sequences present in a very limited fraction of the entire tumour. Therefore, the apparent difference in the tumour and peripheral TCR repertoires could also be due to the high number of intratumour TCR clones that might be present in the periphery but that are not detected due to currently restricted sampling (i.e. single biopsies) used in most studies. Further, rigorous analysis of the potential effect of restricted sampling of total tumour protocols is rarely carried out in published studies to date.

Resolving whether antitumour responses of ICB are (i) due to recruitment of novel T cells, particularly from blood, or (ii) due to functional boosting of pre-existing intratumoural T cells, is relevant to predictive biomarker and cellular therapy development. In the first scenario, longitudinal analysis of the blood TCR repertoire combined with on-treatment tumoural TCR repertoire profiling may be required to predict ICB responders. In the second scenario, analysis of pre-treatment tumoural TCR clonality may be adequate to serve as a baseline predictor of ICB response. Further, if reinvigoration of pre-existing TILs is the dominant mode of action by ICB, harvesting tumour material before treatment followed *ex vivo* enrichment, and expansion of T cells would allow manufacturing of personalised adoptive cellular products.^62^

### Mechanisms underpinning immune adverse reaction in ICB-treated patients

TCR sequencing has also helped to elucidate the mechanism underpinning immune-related adverse events (irAEs) associated with ICB. Luoma et al. carried out scRNA-Seq of colonic tissue from eight healthy individuals and patients with melanoma previously treated with ICB (immune-related colitis present, *n* = 8; absence of colitis, *n* = 6).^63^ Two T-cell transcriptional clusters, representing CD8+ cytotoxic and cycling T cells, were enriched in patients who experienced immune-related colitis. A large percentage of clonally expanded TCR sequences from tissue-resident memory T-cell clusters were detected in the colitis-specific T-cell transcriptional clusters. Therefore, ICB may induce immune-related colitis via the emergence of cytotoxic effector cells from tissue-resident CD8+ T cells present in healthy colon.

Other studies suggest that irAEs could be linked to the establishment of a T-cell response within tumour, in line with data that suggest a correlation between ICB response and toxicity.^64^ A case report of two patients with fulminant myocarditis showed their tumours harboured high-frequency TCR sequences also found at high frequency in cardiac and skeletal muscle.^65^ High levels of muscle-specific antigens, desmin and troponin, in the tumours suggest that cross-reactive TCRs were responsible for the myocarditis in these patients. Another report in 25 patients with NSCLC found that cutaneous lesions from immune-related toxicity shared high-frequency TCR clones with the lung tumour samples, suggesting T cells reacted against shared antigens in the two organs.^66^ However, caveats from this study are the small cohort size and the limited number of patient-matched tumour and skin samples for the TCR clonotype analysis.

Another mechanism that could explain the link between ICB response and irAEs is that a more varied peripheral TCR repertoire can be observed following ICB,^54^ which could increase the chance emergence of *de novo* autoreactive T-cell clones. Supporting this hypothesis, Oh et al*.* found in 21 patients with metastatic prostate cancer that increased diversity of the blood TCR repertoire after anti-CTLA-4 treatment correlated both with clinical response (measured by ≥50% prostate-specific antigen decline) and higher rates of irAEs.^67^

## Conclusions and perspective

TCR sequencing has revolutionised immuno-oncology research; however, several limitations hamper its clinical application. First, current single-cell and bulk TCR sequencing techniques are not standardised or clinically validated and require bespoke computational analyses. Outside of dedicated research settings, the vast majority of the clinically available tumour samples are FFPE samples. These are not suitable for high-quality single-cell TCR sequencing. Finally, a key limitation of TCR sequencing is the inability to determine the antigen specificity of a given TCR sequence; therefore, bespoke *in vitro* T-cell reactivity assays remain indispensable to identify tumour-reactive T cells. We are hopeful that the current intense focus in developing new TCR sequencing technologies and new computational approaches to extract biological information from TCR sequences, including the prediction of the cognate antigen of a given TCR sequence, will help to overcome many of these technical challenges, ultimately for patient benefit.
